# Genetic and Epigenetic Mechanisms That Maintain Hematopoietic Stem Cell Function

**DOI:** 10.1155/2016/5178965

**Published:** 2015-12-20

**Authors:** Christian Kosan, Maren Godmann

**Affiliations:** Center for Molecular Biomedicine (CMB), Department of Biochemistry, Friedrich-Schiller-University Jena, Hans-Knöll-Straße 2, 07745 Jena, Germany

## Abstract

All hematopoiesis cells develop from multipotent progenitor cells. Hematopoietic stem cells (HSC) have the ability to develop into all blood lineages but also maintain their stemness. Different molecular mechanisms have been identified that are crucial for regulating quiescence and self-renewal to maintain the stem cell pool and for inducing proliferation and lineage differentiation. The stem cell niche provides the microenvironment to keep HSC in a quiescent state. Furthermore, several transcription factors and epigenetic modifiers are involved in this process. These create modifications that regulate the cell fate in a more or less reversible and dynamic way and contribute to HSC homeostasis. In addition, HSC respond in a unique way to DNA damage. These mechanisms also contribute to the regulation of HSC function and are essential to ensure viability after DNA damage. How HSC maintain their quiescent stage during the entire life is still matter of ongoing research. Here we will focus on the molecular mechanisms that regulate HSC function.

## 1. Introduction

Hematopoiesis is the development of all mature blood cell lineages that emerge from multipotent hematopoietic stem cells (HSC) in the bone marrow. The human hematopoietic system produces around 10^12^ cells very day. HSC have the ability to differentiate into all hematopoietic lineages but also retain their self-renewal capacity [1]. HSC are located in stem cell niches in the bone marrow that provide signals to maintain stem cell quiescence. Cell intrinsic mechanisms like transcription factor networks and epigenetic regulations have been shown to regulate the balance between self-renewal and differentiation [2]. Under homeostatic conditions HSC cycle very infrequently and stay mainly in G_0_ [3]. This has been shown by two different long-term label-retention assays [4, 5]. These data point to very slow cycling (quiescent) HSC that cycle only every 145 days, which results in about 5 cell divisions per life time [5]. Wilson and coworkers could also show that dormant HSC can be activated by injury and that this is reversible; at least some activated HSC can switch back into a quiescent state. In addition, Takizawa and coworkers could show that life-long multilineage repopulation potential can also be detected in faster cycling cell populations as described for quiescent HSC [4–6]. Interestingly, this faster cycling population can also slow down over time, indicating that divisional activity does not necessarily lead to a loss of HSC function. This contradiction to the work from Foudi and Wilson might be caused by technical differences mainly in FACS-based cell analysis as well as in different* in vivo* tracking systems and different transplantation assays [6]. Furthermore, Takizawa and colleagues could also show that HSC can be efficiently activated using LPS. This is of particular interest to understand how HSC can be activated upon stress.

During differentiation, HSC progressively lose their ability to self-renew and gain lineage specificity of the different hematopoietic lineages [7].

To ensure their life-long functionality, HSC have to be protected against any type of DNA damage. Recent work points to a unique mechanism of how HSC respond to DNA damage (DDR). In quiescent HSC, the response to DNA damage is regulated by a strong induction of p53 and the upregulation of p21, whereas faster cycling multipotent progenitors (MPP) respond with apoptosis [8]. This review focuses on recent findings of how HSC maintain their stem cell capacity by transcriptional regulation as well as epigenetic modifications and, furthermore, how HSC deal with DNA damage upon irradiation and during aging.

## 2. Hematopoietic Stem Cells

The hematopoietic system consists of two major lineages: on the one hand the myeloid lineage and on the other hand the lymphoid lineage. The myeloid lineage includes the cells of the humoral immune response and erythroid cells. The lymphoid lineage consists of B and T cells, the cells of the adaptive immune system, and natural killer (NK) cells. All cellular compartments of the hematopoietic system are derived from hematopoietic stem cells [7]. HSC develop into all hematopoietic lineages following a strict hierarchical order. During this process they gradually lose their self-renewal capacity and gain lineage specificity. Quiescent long-term HSC (LT-HSC) mainly reside in distinct areas of the bone marrow, the so-called stem cell niche [9]. Upon activation LT-HSC leave this niche and migrate towards the blood vessels. Here, they undergo asymmetric cell division, which produces again one LT-HSC and one short-term HSC (ST-HSC) that subsequently differentiates into a multipotent progenitor cell. ST-HSC and MPP still have the potential to differentiate into all hematopoietic lineages but they have lost their self-renewal capacity [10]. Further differentiation into a more committed progenitor is a stepwise process. The common myeloid progenitors (CMP) are restricted to the myeloid lineage and differentiate into granulocyte-monocyte progenitors (GMP) and megakaryocyte-erythrocyte progenitors (MEP). MEP and GMP give rise to erythrocytes and platelets or granulocytes and macrophages, respectively. Lymphoid primed multipotent progenitors (LMPP) give rise to common lymphoid progenitors (CLP), which, in turn, produce progenitors of the lymphoid cells and NK cells. LMPP also have the potential to differentiate into granulocyte-monocyte progenitors (GMP) but have an impaired capacity to develop into cells of the megakaryocytic or erythroid lineages [11–15].

Several signaling pathways have been shown to affect stem cell function like BMP-, Hedgehog-, Notch-, Wnt-, and TGF-*β* signaling pathways. These pathways transduce signals from the microenvironment to activate cell intrinsic signaling cascades to regulate HSC self-renewal, differentiation, apoptosis, senescence, and proliferation. Their function differs with the location of the HSC in the bone marrow niche and the developmental stage and indicates the importance of a tight control of these signaling pathways.

## 3. The Microenvironment of the Bone Marrow-Hematopoietic Stem Cell Niche

Proper HSC function is mandatory to regulate the balanced outcome of all hematopoietic cells and to maintain homeostasis. During adult life HSC are located in the bone marrow niche, where they are in loose contact with stroma cells that regulate the balance of HSC self-renewal and differentiation [9]. The niche provides a complex environment that supports stem cell function by providing cytokines, growth factors, oxygen tension, and nutrients [16–18] and, moreover, the niche is absolutely critical for stem cell quiescence. Distinct cell types in the stem cell niche, like osteoblast and osteoclast, provide this microenvironment for stem cell maintenance.

Several molecular mechanisms have been described to be essential for perpetuation of HSC quiescence. A tight regulation of signaling networks, cytokines and cytokine receptors, adhesion molecules, matrix proteins, and concentration gradients of some chemical molecules are involved in these processes. The hematopoietic stem cell niche is characterized by an intrinsic dynamic to regulate quiescence, self-renewal, and proliferation of hematopoietic stem cells and consists of several cell types that are crucial for HSC quiescence and has been discussed recently [19, 20]. Several chemokines and signaling pathways have been identified to control HSC integrity in the stem cell niche.

**Figure 1:**
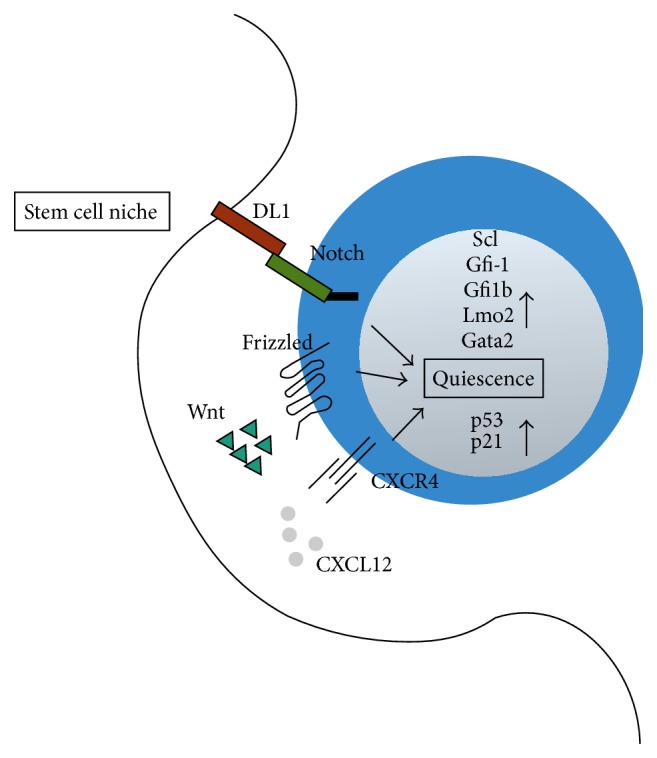
Hematopoietic stem cells maintain quiescence through extrinsic and intrinsic signals. The stem cell niche provides signals that regulate HSC quiescence and localization in the niche. DL1 and Notch; CXCL12 and CXCR4; and Wnt and Frizzled. HSC intrinsic transcription factors regulate signaling process to keep HSC in a quiescent stage.

CXCL12 is a key niche factor that regulates HSC retention in the niche on the one hand and HSC quiescence and multilineage potential [21]. Deletion of CXCR4, the receptor for CXCL12, results in a substantial loss of HSC and demonstrates an essential role of CXCL12-CXCR4 signaling in maintaining HSC quiescence [22]. Furthermore Notch- and Wnt/*β*-catenin signaling as well as TGF-*β* signaling pathways are discussed to be important regulators of HSC function. For example, Wnt/*β*-catenin signaling provides essential signals to ensure quiescence and preserves self-renewal capacity [23]. However, inactivation of *β*-catenin and *γ*-catenin does not affect HSC function or hematopoiesis in general (Figure 1) [24, 25].

There are some controversial findings, which might result from different experimental approaches and the complexity of the signaling pathways. This topic is extensively discussed in an excellent review from Mendelson and Frenette [19].

## 4. Transcription Factor Networks in HSC

During development, HSC follow specific differentiation programs that are distinct for embryonic and adult development [26]. Transcription factors play a key role in regulating these differentiation programs besides maintaining stem cell quiescence in the adult organism. In order to meet different requirements, these transcription factors act in huge regulatory complexes, often with different binding partners. However, there are about 50 different transcription factors that have been shown to affect HSC functionality and behavior throughout adult life. Interestingly, many of these transcription factors are imbedded in regulatory networks that show a very high degree of connectivity. In this section, we will just focus on a selection of key transcription factors that are required for HSC production, survival, and self-renewal.

Several key transcription factors that regulate HSC function have been identified by gene targeting experiments (Figure 1) [27]. But due to the different requirements during fetal and adult hematopoiesis and different knockout and experimental strategies the results obtained from these experiments are sometimes not easy to interpret. One key regulator of hematopoiesis is the helix-loop-helix transcription factor Scl/Tal1. The complete ablation of Scl is embryonic lethal and points towards a defect in fetal HSC genesis [28, 29]. Different conditional knockout studies shed more light on the function of Scl in the hematopoietic system: Scl is essential for HSC development already at early embryonic development in the yolk sac and remains indispensable for proper megakaryocyte and erythroid development in the adult mouse [30, 31]. Furthermore, Scl is also required for the regulation of quiescence and long-term potential of HSC. Surprisingly, Scl is not essential for self-renewal and multipotency of these cells [32]. In this study, Scl was conditionally inactivated only in the adult mouse using an inducible Mx-Cre deleter pointing to a dominant role of Scl during embryonic development. Supporting this hypothesis, Schlaeger and coworkers could show in a Tie2-Cre-mediated knockdown that Scl is only required in a very tight window during fetal development [33]. Nevertheless, a more recent study showed that a reduced level of Scl impairs HSC function and that Scl/Tal1 is also required for the regulation of HSC quiescence and the long-term potential of hematopoietic stem cells in the adult mouse [34]. These contradictive results might be explained by different experimental setups; further studies have to bring insights about the exact role of Scl in adult hematopoiesis.

However, when Scl is expressed together with LIM only protein 2 (Lmo2) and GATA binding factor 1 (Gata1), SCL induces ectopic blood cell development, which, again, underlines the importance of Scl in HSC genesis [35, 36]. The transcription factor Lmo2 regulates the formation of DNA-binding complexes, although Lmo2 itself does not bind directly to the DNA [37]. Gata2 regulates development as well as cell cycle progression and proliferation of hematopoietic stem and progenitor cells. Gata2 (Gata2^−/−^) knockout mice are embryonic lethal and Gata2^−/−^ ES cells have been shown to be perturbed in the production of hematopoietic cells in chimeric mice. Moreover, HSC showed reduced responsiveness to stem cell factor (SCF) and increased apoptosis [38–40].

Using conditional mouse models it has been shown that Gata2 is required for the generation of HSC at the stage of endothelial-to-hematopoietic cell transition and for HSC survival [38]. It has previously been demonstrated that Gata2 interacts with Runx1, to regulate transcription of genes relevant to hematopoietic cell development and growth [41, 42].

Interestingly, experiments done by Chen and coworkers were performed with the same Cre-recombinases (Vec-Cre and Vav1-Cre) as they have previously been used to generate a conditionally inactive form of Runx1 [43]. This revealed that the previously described concurrent function of Gata2 and Runx1 is only essential in endothelial-to-hematopoietic transition (EHT) but also revealed important new insights into separate GATA2 and RUNX1 functions in hematopoietic progenitors and HSC at different developmental stages [38]. Mice deficient for Gata1 die at embryonic stages E10.5–E11.5 due to a lack of red blood cells (RBCs) [44]. In contrast to Gata2, Gata1 is more restricted to the erythroid and megakaryocytic lineage and HSC are almost negative for Gata1 expression but ectopic expression of Gata1 in HSC resulted almost exclusively in MegE-affiliated colonies [45]. This clearly points towards a lineage determining role rather than a function in HSC maintenance.

Other major regulators crucial for maintaining HSC functions are the zinc finger transcription factors growth factor independent 1 (Gfi1) and growth factor independent 1b (Gfi1b). Gfi1 is expressed in several hematopoietic cells types, like myeloid and lymphoid cells, and Gfi1 is also expressed in HSC. Gfi1 (Gfi1^−/−^) deficiency leads to severe anemia [46–50].

Hock and coworkers detected at least as many, if not more, HSC in the bone marrow of Gfi1-deficient mice compared to WT mice [51]. In contrast, the group of Tarik Möröy observed strong reduction of HSC in Gfi1^−/−^ mice [50]. This might be due to different gating strategies in the FACS analysis, since both knockout strategies are pretty similar. This illustrates the difficulty and complexity of the analysis of very small cell populations like HSC.

HSC derived from Gfi1^−/−^ mice show a reduced self-renewal capacity and a perturbed repopulation capacity after adaptive transfer in syngeneic mice [50, 51].

A larger number of HSC in Gfi1-deficient mice were in the proliferative stages of the cell cycle, indicating that Gfi1 functions to restrain HSC proliferation. In conclusion, both groups suggest that the excessive proliferation of Gfi1^−/−^ HSC results in exhaustion and this leads to loss of self-renewal capacity. Consistent with this was a downregulation of the negative cell cycle regulator p21^Cip^, which is required to maintain HSC in G_0_ [51]. However, recent reports point more to a role of p21^Cip^ in regulating cell cycle activity during stress response rather than homeostasis (see cell cycle section) [4, 52]. That might indicate that p21^Cip^ downregulation is not the sole reason for the phenotype in Gfi-1^−/−^ mice. Moreover, it has been shown that Gfi1 fulfills an additional role in HSC maintenance by protecting HSC from apoptosis [53].

The transcription factor Gfi1b is indispensable for embryonic and adult erythroid development, and it is highly expressed in HSC [48, 54, 55]. Conditional inactivation of Gfi1b using an inducible Mx-Cre line leads to an increased frequency of HSC but a reduced quiescence [53]. However, multipotency and self-renewal capacity are not altered in these mice [53].

HSC from Gfi1b deficient mice show a reduced expression of adhesion molecules, like CXCR4 and the vascular cell adhesion protein-1 (Vcam-1), which are required to retain HSC in the stem cell niche [48].

All in all, both transcription factors, Gfi1 and Gfi1b, are essential for HSC maintenance and preserve quiescence, due to different molecular mechanisms.

The importance of a proper interaction of HSC with their niche is once more demonstrated by aberrant expression of the transcription factor c-Myc. Ectopic expression of c-Myc in HSC leads to a loss of self-renewal capacity and induces lineage differentiation due to repression of N-cadherin and integrins. These data point to an essential role of c-Myc in regulating the balance between stem cell self-renewal and differentiation, most likely by influencing the interaction of HSC with the stem cell niche [2, 56].

Just to name a few more examples, Ets transcription factors like Pu.1, Erg, and Fli1, homeobox factors like HoxA9 and HoxA10, the TALE family transcription factor Meis1, and Helix-loop-Helix proteins like E2A are other essential transcription factors that are involved in HSC development and maintenance. Their functions have been extensively discussed in a recent review [57]. The rising number of factors involved in HSC fate and function and recent findings that these factors act in distinct complexes add another level of complexity, which requires different approaches to get insight into the underlying molecular mechanisms. Wilson and coworkers used a Chromatin Immunoprecipitation-Sequencing (ChIP-Seq) approach to identify genome-wide binding sites of ten key hematopoietic transcription factors (Scl/Tal1, Lyl1, Lmo2, Gata2, Runx1, Meis1, Pu.1, Erg, Fli-1, and Gfi1b) [42]. Using a multifactor ChIP-Seq approach has the advantage of a more comprehensive view of regulatory mechanism. The authors could identify new complexes directly bound to regulatory elements that are essential for specific processes in HSC. Furthermore, the authors could confirm that Runx1, Gata2, and Scl control a set of genes that are critical in regulating the balance between quiescence and self-renewal of HSC [42]. These findings give new insights into the role of each factor in its transcriptional network and might also explain the compensatory effect of some factors regulating HSC function.

To activate or repress gene expression, transcription factors recruit cofactors to their binding sites to regulate accessibility of regulatory regions. For example, Gfi1 and Gfi1b recruit CoREST and LSD1 to the promoter regions of target genes to mediate transcriptional repression [58]. Furthermore, Gfi1 and Gfi1b also recruit histone deacetylases (HDAC) to promoter regions of target genes to downregulate transcription [59–61]. This clearly points to a crucial role of epigenetic mechanisms in sustaining HSC quiescence and changes in epigenetic patterns might lead to changes of the genetic program and eventually HSC fate. This will be discussed in more detail in the next section.

## 5. Epigenetic Modification in HSC

Somatic cells of an individual share per se the same genetic information. What puts a face on cells and evolves them into true specialists to fulfill different body functions is an additional layer of so-called epigenetic information that is imposed on the DNA or histone proteins, for example, DNA methylation and posttranslational histone modifications. These epigenetic modifications eventually result in a defined chromatin structure and thus an “individual” gene expression pattern in a cell [62].

In the healthy adult body, only lineage-restricted, hence multipotent or unipotent, stem cells can be found. These cells are already equipped with specific sets of epigenetic marks that provide the basic information for directions of subsequent differentiation processes. The hematopoietic system provides an ideal model system to study the correlation between epigenetically directed changes in chromatin structure and gradual restriction of cell potential during differentiation, since the hematopoietic cell lineage development follows a strict hierarchical pattern emanating from a single primary source, the hematopoietic stem cell. HSC are multipotent and have the ability either to self-renew to maintain the HSC pool throughout life or to differentiate into all functional blood cells. The molecular mechanisms that actually determine hematopoietic cell fate or lineage commitment are highly complex and are not well understood today. Global gene expression analyses in combination with epigenetic profiling of hematopoietic cells at different developmental stages will provide a more comprehensive picture of cell fate decisions in hematopoiesis and thinking one step further in disease conditions. Here, the unanswered question is: What happens if the highly orchestrated establishment of epigenetic signatures in differentiating cells is disturbed?

### 5.1. Histone Methylation in HSC

Covalent histone modifications are crucial for virtually all cellular processes that modulate the access to the genomic DNA, like transcription, DNA replication, DNA repair, meiosis, or compaction of DNA. They directly influence the structure of nucleosomes or generate a signaling platform to recruit so-called “reader” proteins that mediate downstream effects. Over 100 different histone modifications have been identified to date, with more being expected to be discovered in the near future (for review see [62, 63]).

One classical posttranslational histone modification mark is the methylation of lysine or arginine residues. Histone methylation is a reversible process: methyl marks are established by histone methyltransferases, so-called “writers,” and removed by histone demethylating enzymes, termed “eraser” [64]. Thus, a great variety of histone methylation states is created and different methylation states are linked to different biological processes. The role of histone methylation states in fine-tuning gene expression has been well studied and key findings in cell fate decisions have been made in embryonic stem cells (ESC) as well as in HSC [65–67]. Here, we focus on recent advances that link regulation of gene expression in HSC fate decisions to histone methylation events that occur on histone H3 lysine 4 (H3K4), histone H3 lysine 9 (H3K9), and histone H3 lysine 27 (H3K27).

A phenomenon firstly described in embryonic stem cells (ESC) is the appearance of bivalent chromatin, which refers to distinct gene regulatory regions that are simultaneously occupied with counteracting marks. Mostly, key developmental genes that are poised in stem cells carry a bivalent epigenetic signature, namely, repressive H3K27me3 and activating H3K4me3 marks, in order to keep these genes quiet but to enable quick activation once a path of differentiation has been taken [68]. Upon initiation of differentiation bivalent chromatin structures are resolved and poised genes are either activated by removing repressive H3K27me3 marks or permanently silenced by loss of activating H3K4me3 marks and accumulation of repressive H3K27me3 marks [68, 69]. Bivalent chromatin is also found in human and murine HSC. Genome-wide high resolution mapping of histone modifications revealed an association of developmental genes with bivalent epigenetic signatures in HSC and precursor cells, and, upon lineage commitment and differentiation, these bivalent structures are also resolved [65, 70]. Interestingly, genes that become activated upon resolution of bivalent epigenetic signatures and in the course of lineage commitment are additionally associated with H3K4me1 and H3K9me1 marks in gene regulatory regions and also bound by RNA Pol II. This indicates that the choice of resolution of bivalent signatures during differentiation is already predetermined in HSC or progenitor cells [65].

H3K4 methylation is established by the SET1 and mixed lineage leukemia (MLL) family of histone methyltransferases [71–73] and is removed by the lysine-specific demethylase 1 (LSD1) [74] and the Jumonji AT-rich interactive domain 1 (JARID1) family of histone demethylases [75] (for review see [76]). The writer of repressive H3K27 methylation marks is the histone methyltransferases enhancer of zeste homolog 2 (EZH2), a subunit of the polycomb repressor complex 2 (PCR2) [77, 78], and erasers of methylated H3K27 include histone demethylase UTX (also known as Kdm6a) and Jmjd3 (also known as Kdm6b) [79–81].

The polycomb repressor complexes have been well characterized in HSC and are generally recognized as key factors in lineage determination. PRC2 mediates gene repression through the enzymatic activity of its histone methyltransferase subunit EZH2, which catalyzes H3K27 methylation. Methylated H3K27 is a binding platform for PRC1, which, in turn, is involved in heterochromatin formation. Genetic ablation of BMI1, a component of the PRC1, results in defective long-term self-renewal capacity of HSC and in severe postnatal pancytopenia [82, 83]. Moreover, loss of BMI1 leads to accelerated lymphoid specification due to premature expression of* Ebf1* and* Pax5* lineage markers, which are marked with bivalent epigenetic modifications in wild-type HSC [84]. In contrast, genetic deletion of* Ezh2*, the catalytic subunit of the PRC2 complex, does not compromise HSC self-renewal and H3K27 methylation is retained, probably due to complementary activity of Ezh1 [85, 86]. Interestingly, ectopic expression of Ezh2 causes a significant increase in HSC numbers and myeloid lineage cells in a knock-in mouse model [87].

Another enzyme that actively shapes a cell's epigenome and eventually a cell's fate is the lysine-specific demethylase 1 (LSD1). LSD1 specifically removes one or two methyl groups from histone H3 lysine 4 (H3K4) or 9 (H3K9), depending on the cellular context and the presence of cofactors, thereby repressing or activating transcription [74, 88]. The complete LSD1 knockout results in embryonic preimplantation lethality and embryonic stem cells lacking LSD1 activity fail to differentiate fully [89–91]. Conditional ablation of the lysine-specific histone demethylase LSD1 in murine hematopoietic stem cells results in an aberrant accumulation of LSD1 substrates, namely, activating H3K4me1 and H3K4me2 marks, at enhancers and promotors of stem- and progenitor cell-specific genes in differentiating cells. Failure to repress early lineage gene expression impairs hematopoietic maturation programs and eventually results in profound multilineage hematopoietic differentiation defects. This conversely means that, upon differentiation, LSD1 removes activating H3K4 methylation marks (H3K4me1, H3K4me2) from enhancer and promoter regions in order to silence hematopoietic stem and progenitor cell-specific genes and to promote differentiation [92].

### 5.2. DNA Methylation

Context- and tissue-specific gene expression is influenced by DNA methylation. The DNA methyltransferases DNMT3a and DNMT3b are responsible for* de novo* methylation of CpG dinucleotides whereas DNMT1 preserve DNA methylation patterns after DNA replication [93–95]. There is a remarkable plasticity in DNA methylation signatures during hematopoiesis pointing towards an essential role of methylation changes in cell fate and lineage commitment. Ji and colleagues analyzed differential methylation patterns in purified cell populations, hierarchically progressing in development and with well-characterized differentiation potentials. They developed a comprehensive map that indicates an orchestrated modulation of the DNA methylome during myeloid and lymphoid commitment from haematopoietic progenitors and that DNA methylation signatures specifically vary depending on the branch of differentiation, either myelopoiesis or lymphopoiesis, progenitors choose [96]. In principle, genes, involved in maintaining a more undifferentiated state, were progressively methylated and transcriptionally silenced in stem cells or progenitors as development proceeded. The transcription factors Meis1, Hoxa9, and Prdm16 were among these candidates [96–99]. On the other hand, genes that are initially transcriptionally inactive and found methylated in HSC or progenitors experience a selective demethylation during lineage commitment. For example, myeloid specification from MPP (multipotent progenitor) through GMP (granulocyte macrophage progenitor) cells was accompanied by transcriptional upregulation and progressive hypomethylation of Mpo, which encodes an enzyme central to the microbicidal activity of neutrophils [100, 101]. Moreover,* Gadd45a*, which is implicated in myeloid development, was found to be concomitantly upregulated and demethylated in the CMP to GMP transition [96]. The underlying mechanisms of (a) gene-specific DNA* de novo* methylation and (b) selective DNA demethylation in hematopoiesis, for example, passive dilution of 5mC in absence of DNMTs or active removal of modified 5mC via base excision repair (BER) mechanisms [102], are not well understood so far.

**Figure 2:**
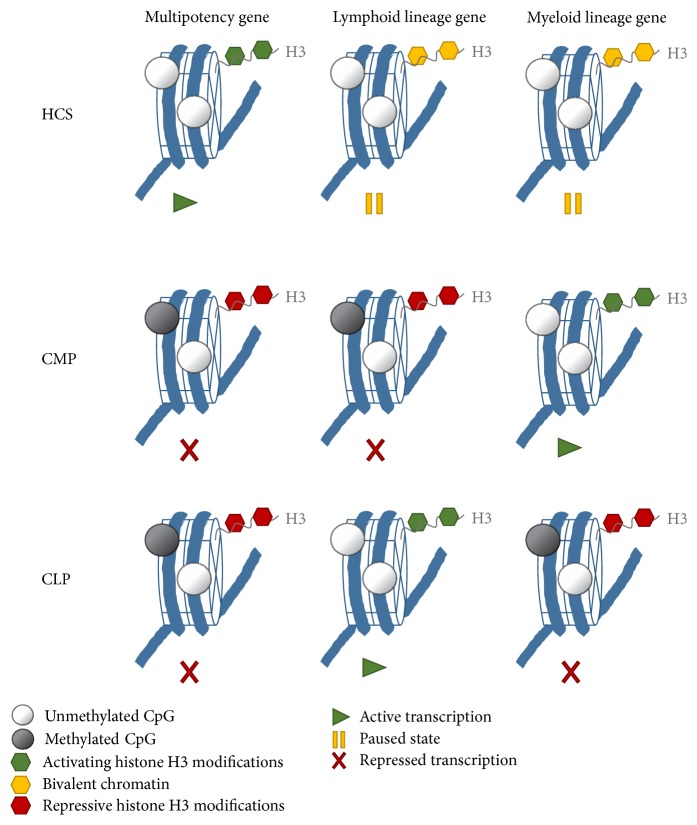
Changes in epigenetic signatures upon HSC differentiation and lineage commitment. Multipotency genes are actively transcribed in hematopoietic stem cells (HSC) to maintain stemness and self-renewal. Promoter and enhancer regions are labelled with activating H3K4 methylation marks (green hexagons) and unmethylated CpG islands (white circles). Key genes, important for lineage commitment and differentiation into progenitor cells of the myeloid (common myeloid progenitors (CMP)) or lymphoid lineage (common lymphoid progenitors (CLP)), are kept in a paused state mainly by the counteracting histone methylation marks H3K27me3 (repressive) and H3K4me3 (activating) in gene regulatory regions, termed as bivalent signatures (yellow hexagons). Upon lineage commitment, multipotency genes are silenced by repressive histone methylation marks (e.g., H3K27m3, H3K9me3, and red hexagons) and partially by gene-specific* de novo* methylation of CpG islands (grey circles). Upon differentiation bivalent chromatin signatures are resolved depending on lineage choice: paused genes become either activated transcriptionally by accumulation of activating H3K4me3 marks and loss of repressive H3K27me3 or silenced by loss of H3K4me3 and accumulation of H3K27me3 and partially by gene-specific methylation of CpG islands.

Conditional knockout mouse models try to explain the role of DNA methylation in maintenance of the HSC pool versus lineage commitment. HSC-specific deletion of the maintenance DNA methyltransferase revealed an essential function for DNMT1 in HSC self-renewal, niche retention, and proper differentiation of the myeloid lineage. DNMT1 seems to be critical for HSC and progenitor cell state transitions, such as the stepwise differentiation of HSC to ST-HSC/MPPs and ST-HSC/MPPs to myeloid progenitor [103]. Conditional inactivation of one or both* de novo* DNA methyltransferases DNMT3a and DNMT3b in HSC underlined the importance of gene-specific* de novo* methylation in regulation of HSC fate decisions [104, 105]. Loss of Dnmnt3 progressively impairs HSC differentiation over serial transplantation, while, at the same time, HSC numbers in the bone marrow increase. Methylome analyses of differentiating HSC descendants revealed a reduction in global as well as a loci-specific DNA methylation and consequently an increase in HSC multipotency genes, for example, Runx1 and Gata3. In conclusion, DNMT3 plays a key role in HSC fate by repressing the HSC program and triggering lineage commitment. DNMT3 presumably silences HSC-specific genes by* de novo* DNA methylation and thus enables differentiation [104]. Combined loss of Dnmt3a and Dnmt3b was synergistic, resulting in enhanced HSC self-renewal and a more severe block in differentiation than in Dnmt3a-null cells, whereas loss of* Dnmt3b* resulted in a mild phenotype [105]. Epigenetic regulation of lineage-specific genes is a signature of HSC differentiation and lineage commitment (Figure 2).

Due to different requirements of HSC at different developmental stages, HSC show a difference in cell cycle activity. During fetal life HSC produce homeostatic levels of blood cells, mainly erythroid cells, to supply the fast growing organism with sufficient oxygen. In line with this, almost 100% of all fetal HSC are constantly cycling [106, 107]. During late stages of fetal development and the first 2-3 weeks of neonatal life HSC migrate to their bone marrow niche and adult HSC are considered to be quiescent. This does not happen immediately after seeding the niche, but at 4 weeks of age 95% of HSC are quiescent. How this is achieved is not fully understood, yet. By this time, the development of the bone marrow niche is completed and perhaps feedback mechanisms of blood cells that have reached homeostasis are established [106, 108, 109].

## 6. Cell Cycle Regulation in Hematopoietic Stem Cells

One key mechanism to maintain quiescence under homeostatic conditions in HSC is the control of cell cycle regulators (for review see [110]). Cyclins and Cyclin-dependent kinases (Cdks) regulate cell cycle progression. Cdks are serine-threonine kinases and after binding cyclins Cdks are recruited to their target proteins that regulate them through phosphorylation. Cyclin D1, cyclin D2, and cyclin D3 are expressed at different levels in HSC [111]. Quiescent HSC are in G_0_. To enter the cell cycle HSC have to activate the cyclin D/Cdk4/6 complex that ensures entry into G_1_ by repression of the Retinoblastoma (RB) protein via phosphorylation. Inactivation of RB leads to the entry of S-phase by the activation of E2F transcription factors. Whether or not HSC enter the cell cycle is controlled by external signals like growth factors provided by the niche. If cells pass the “restriction point” between early and late G1 phase, they are determined to pass through the whole cell cycle. This makes G1/S transition very critical to regulate HSC quiescence. Inactivation of one of these factors has only a small effect on stem cell function pointing to a redundant role during cell cycle regulation [112, 113]. Nevertheless, D1/2/3-cyclins^−/−^ mice and Cdk4/6^−/−^ mice have a decreased number of HSC in the fetal liver and reduced proliferation in erythroid cells [113, 114].

A recent study showed that CDK6 levels are essential for cells leaving G_0_ and that HSC in the absence of CDK6 cannot respond to mitogenic signals [115, 116]. Interestingly, quiescent LT-HSC and ST-HSC show different levels of CDK6. Quiescent LT-HSC do not express CDK6 and this delays cell cycle entry by 5-6 hours. In contrast, ST-LSK express moderate levels of CDK6 to keep them in a quiescent state and to allow them to enter the cell cycle upon mitogenic stress [115]. This delayed G_0_ exit might also be an important mechanism to coordinate DNA-repair of LT-HSC (see DNA damage response in HSC).

Negative cell cycle regulators interact with Cdks and lead to a cell cycle arrest and this makes CDK potent candidates to sustain stem cell quiescence by preventing cell cycle entry. They can be subdivided into INK4-family members (p15, p16, p18, and p19) binding to Cdk4/Cdk6 and Cip/Kip-family members (p21, p27, and p57) binding Cdk4/Cdk6 and Cdk2.

The negative cell cycle regulator p57^Kip1^ is highly expressed in quiescent hematopoietic stem cells and declines with ongoing differentiation [111, 117]. Conditional inactivation of p57 using an inducible Mx-Cre line (active in all hematopoietic cells) leads to a decrease of quiescent HSC and a perturbed capacity to reconstitute irradiated syngeneic mice [118]. Following a different approach, Zou and coworkers used fetal liver (FL) cells for transplantation experiments, because p57^−/−^ mice are neonatal lethal, to investigate the role of p57^Kip1^. They observed a normal reconstitution of the hematopoietic system after the first and second round of transplantation and only a mild decrease after the third round of transplantation [119]. The authors noticed a significant compensatory upregulation of p18 and p27 in p57^−/−^ FL HSC. Inactivation of p27 in p57^−/−^ FL HSC leads to a reduced reconstitution capacity after serial transplantation and decreased HSC quiescence. This indicatesthat the compensatory upregulation of p27 in p53-deficient FL HSC preserves HSC functionality and quiescence. In addition, inhibition of p27 alone does not affect the number of self-renewing HSC but however increases the size of the haematopoietic progenitor pool [120]. Taken together, these findings show that p27 can compensate for the loss of p57 but also points to distinct role of some Cdk inhibitors during hematopoietic development under normal conditions.

The negative cell cycle regulator p21^Cip^ is highly expressed in HSC and a role in regulating quiescence had been suggested, since p21-deficient (p21^−/−^) mice show an increased proliferation and a higher number of HSC [121]. However, recent reports point to a role of p21^Cip^ in regulating cell cycle activity during stress response rather than during homeostasis [4, 52]. This finding was further supported by p57 and p21 deficient HSC. These mutants resembled p57 deficient HSC and only showed a severe reduction in colony-forming capacity, suggesting again an important role of p21 during stress response rather than during homeostasis [118].

It has recently been shown that also the members of the INK4-family play a critical role in HSC functions. p16^INK4a^ expression is repressed by EZH1 in young mice [122] but increases with age [123]. This leads to a decreased self-renewal, homing, and repopulating of HSC in response to stress [123]. Nevertheless, maintaining steady-state HSC aging* in vivo* appears to be independent of p16^INK4a^ [124]. Mice missing the negative cell cycle regulator p19^INK4d^ show a reduced number of HSC and an increased G_0_/G_1_ transition [125]. Furthermore, p19^INK4d^ deficient HSC showed a decreased transition through S/G2-M phase and an increase in apoptosis [125].

## 7. DNA Damage Response in HSC

During their entire life hematopoietic stem cells are mainly quiescent and this is considered as a protective mechanism to minimize cell intrinsic stress [126]. Mice lacking proper DNA damage repair (DDR) mechanisms show severe hematopoietic phenotypes caused by malfunctioning HSC [3, 127, 128]. In particular, HSC accumulate DNA damage during aging, which is responsible for the development of cancer in elderly people [3]. Defects in DDR have also been associated with a wide range of human blood diseases [129]. It has previously been shown that HSC are more radioresistant than faster cycling progenitor cells [130, 131]. This is consistent with intracellular levels of reactive oxygen species (ROS) in HSC and developing progenitors [132]. Quiescent HSC showed a reduced intracellular ROS concentration compared to other progenitor cells [132]. Interestingly, ROS levels of HSC are regulated by Forkhead transcription factors. FoxO1, FoxO3, and FoxO4 deficient mice showed elevated ROS levels in HSC, whereas ROS levels in myeloid cells were not altered, indicating that FOXO-proteins act specifically in quiescent HSC to maintain their functionality [132].

**Figure 3:**
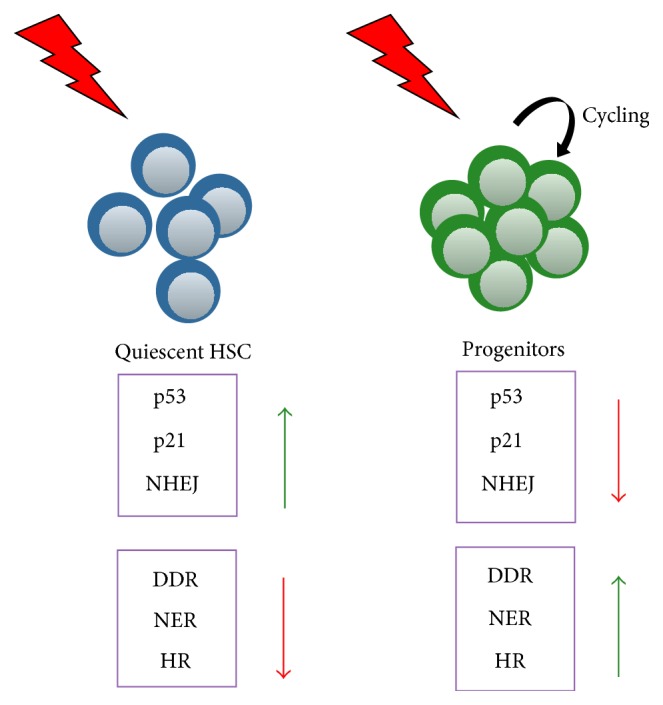
Quiescent HSC and proliferating progenitors respond differently to DNA damage (radiation). HSC show a higher expression level of p53 and p21 and they activate the error-prone repair pathway compared to faster cycling progenitors. They show a reduced DDR, NER, and HR response. (NHEJ: nonhomologous end joining; DDR: DNA damage response; NER: nucleotide excision repair; and HR: homologous recombination).

Hematopoietic stem and progenitor cells (HSPC) from young are more resistant to radiation than downstream myeloid progenitor cells [8]. This radiation resistance is mediated through “ataxia telangiectasia mutated” (ATM) because in the absence of ATM all populations are equally sensitive to radiation. The authors could further show that quiescent HSC induce cell cycle arrest, the expression of prosurvival factors, and the activation of concurrent DNA repair. Upon stress, HSC also induce a strong p53 response and an upregulation of proapoptotic genes (i.e., bax, nova, and puma) and Cdkn1a (p21) expression. In contrast, myeloid progenitors are mainly eliminated by apoptosis [8]. HSC preferentially show nonhomologous end joining- (NHEJ-) mediated DNA repair that renders quiescent HSC susceptible to genomic instability, which can contribute to HSC loss of function and malignant transformation. In contrast, proliferating HSC undergo DNA repair using homologous recombination (HR) mechanism and show a decreased risk of acquiring mutation (Figure 3) [8].

Interestingly, a recent study demonstrated a unique but different mechanism of how human HSC respond to DNA damage. Here, DNA damage is regulated by a strong induction of p53-induced apoptosis [133]. p53-induced apoptosis in human umbilical cord blood HSC can be inhibited by blocking p53 expression or ectopic expression of the antiapoptotic factor Bcl-2. Interestingly, Milyavsky and coworkers also showed that p53 fulfills an apoptosis-independent role in the regulation of self-renewal capacity of HSC [133]. To investigate the role of p53 in long-term repopulation the authors performed serial transplantation assays and could show that inactivation of p53 leads to an accumulation of DNA damage and a decreased self-renewal capacity compared to Bcl-2 overexpressing cells. This clearly points to an apoptosis independent function of p53 in long-term repopulation experiments. Why murine HSC respond with DNA repair and do not undergo apoptosis is not fully understood yet. However, there are also some technical differences in these two studies. First of all, umbilical cord blood HSC are not equivalent to quiescent murine HSC. In addition, transplantation efficiency of human HSC in mouse bone marrow niches might not provide the same homing capacity and a decreased survival rate.

Mohrin and colleagues postulated that also high levels of prosurvival factors counteract the proapoptotic signals. Furthermore, the transcription factor Myc interacting zinc finger protein 1 (Miz-1) has been shown to be the “Miz-ing” link in switching from life to death [134, 135]. Miz-1 regulates Bcl-2 expression and Miz-1 deficient mice showed a high degree of apoptosis in the hematopoietic system [136, 137]. p53 is highly expressed in murine HSC and regulates HSC quiescence and self-renewal and this is critical for preserving the lifelong HSC pool [138]. p53-deficient mice show an increased percentage of HSC, with a reduced potential of long-term repopulation [138, 139]. Furthermore, p53 reduces also the level of ROS under physiological conditions. The high level of p53 in quiescent HSC protects stem cells from DNA damage and accumulation of mutations [140].

Aging of the hematopoietic system is associated with several changes like reduced lymphocyte counts and antibody diversity, autoimmunity, altered regeneration potential, and finally diseases like leukemia [141–143]. It has been shown that the HSC pool changes and lymphoid primed HSC decline with age, while myeloid primed HSC increase [144]. However, also myeloid progenitors lose their functionality [145]. Furthermore, the decreased potential of aged HSC might be caused by an age-associated accumulation of DNA damage [3, 142, 146] or it might be also linked to telomere shortening [147, 148]. Very recent findings show that replicative stress leads to DNA damage in old HSC. In addition, persistent damage was connected to a reduced expression of rDNA genes. This leads to fewer ribosomes and subsequently a reduced protein synthesis [149].

These findings point to a specific mechanism in quiescent HSC to protect them against DNA damage. Thus, there is a necessity that quiescent HSC have to be protected against DNA damage during aging to maintain functionality. Otherwise, they would either lose their stem cell potential or acquire mutations that might lead to severe hematopoietic disorders.

However, recent findings demonstrate an attenuation of DNA damage responses during aging that leads to an accumulation of damaged DNA in quiescent HSC [3, 150]. Aging HSC acquire DNA damage over time, and if these cells are activated by stress signals, they show a severely reduced functional capacity. This explains why aged HSC lose their potential to generate different hematopoietic lineages [141, 151]. Furthermore, a recent study also showed that DNA damage is a direct consequence of HSC to leaving their homeostatic quiescence in response to physiological stress, like infections or anemia [152].

Interestingly, recent work shows that repair mechanisms are activated upon entering cell cycle [153]. Beerman and colleagues could show that quiescent HSCs and progenitor cells acquire similar amounts of DNA damage but attenuation of the DNA repair and response pathways lead to an accumulation of damaged DNA in HSC over time. Cell cycle entry of these HSC leads to the activation of multiple DNA damage response pathways and the repair of accumulated DNA damage [153]. Together with the finding that activated HSC can return into quiescence this might be a possibility of how DNA damage repair mechanisms can maintain the stem cell pool, at least for some HSC [5].

## 8. Conclusion

In this review, we discussed several factors that contribute to the functionality of the hematopoietic stem cell compartment, with special regard to maintenance of stemness versus lineage commitment and differentiation. Several transcription factors, epigenetic modifiers, and cytokine and chemokine signals are crucial to balance cell fate in hematopoiesis. During aging, these mechanisms might lose effectiveness, since the HSC pool shows a reduced capacity in replenishing the hematopoietic system with mature cells and an altered lineage potential [142, 154, 155]. Understanding these molecular mechanisms is of tremendous importance, not only to obtain new insight into developmentally related cell fate decisions but also to gain knowledge about the origin of hematopoietic diseases like cancer, anemia, or autoimmunity. Moreover, this knowledge might eventually also contribute to improvement of the efficiency in adaptive transfer of HSC in immunodeficient patients or after radiation therapy.
